# The PIKfyve Inhibitor YM201636 Blocks the Continuous Recycling of the Tight Junction Proteins Claudin-1 and Claudin-2 in MDCK cells

**DOI:** 10.1371/journal.pone.0028659

**Published:** 2012-03-01

**Authors:** Joseph D. Dukes, Paul Whitley, Andrew D. Chalmers

**Affiliations:** Department of Biology and Biochemistry, Centre for Regenerative Medicine, University of Bath, Bath, United Kingdom; Northwestern University Feinberg School of Medicine, United States of America

## Abstract

Tight junctions mediate the intercellular diffusion barrier found in epithelial tissues but they are not static complexes; instead there is rapid movement of individual proteins within the junctions. In addition some tight junction proteins are continuously being endocytosed and recycled back to the plasma membrane. Understanding the dynamic behaviour of tight junctions is important as they are altered in a range of pathological conditions including cancer and inflammatory bowel disease. In this study we investigate the effect of treating epithelial cells with a small molecule inhibitor (YM201636) of the lipid kinase PIKfyve, a protein which is involved in endocytic trafficking. We show that MDCK cells treated with YM201636 accumulate the tight junction protein claudin-1 intracellularly. In contrast YM201636 did not alter the localization of other junction proteins including ZO-1, occludin and E-cadherin. A biochemical trafficking assay was used to show that YM201636 inhibited the endocytic recycling of claudin-1, providing an explanation for the intracellular accumulation. Claudin-2 was also found to constantly recycle in confluent MDCK cells and treatment with YM201636 blocked this recycling and caused accumulation of intracellular claudin-2. However, claudin-4 showed negligible endocytosis and no detectable intracellular accumulation occurred following treatment with YM201636, suggesting that not all claudins show the same rate of endocytic trafficking. Finally, we show that, consistent with the defects in claudin trafficking, incubation with YM201636 delayed formation of the epithelial permeability barrier. Therefore, YM201636 treatment blocks the continuous recycling of claudin-1/claudin-2 and delays epithelial barrier formation.

## Introduction

Cell-cell contacts between epithelial cells are mediated via different types of specialised junctional complexes, including tight junctions, adherens junctions and desmosomes [1], [2], [3]. The most apical of these complexes, the tight junctions, are composed of transmembrane proteins including claudin family proteins, occludin like proteins, junctional adhesion molecules (JAMs) and plaque proteins such as ZO-1 [4]. Tight junctions control the paracellular flux of molecules across epithelial sheets and selectivity is determined by claudin composition [5].

Despite providing an efficient barrier to permeability it appears that tight junctions are not static structures [4]. Junctional components can be rapidly altered by an array of environmental, physiological and cell cycle-dependent stimuli [6], [7]. These include TNFα which drives the internalization of tight junction proteins such as occludin [8], thus altering barrier function. In addition to stimulus induced endocytosis, there is mounting evidence that tight junctions show dynamic behaviour in unstimulated epithelial monolayers [4]. There is movement of individual tight junction proteins within the tight junctions [9] and claudin-1 is constantly endocytosed and recycled back to the plasma membrane in a range of epithelial cell lines [10]. Occludin also constantly recycles in some epithelial cell lines, but not in MDCK cells [10], [11]. Understanding how these dynamic events are involved in the formation, maintenance and modulation of tight junctions is important as changes in tight junctions have been linked to a wide range of pathological conditions including inflammatory bowel diseases and cancer [6], [7], [12], [13], [14].

Our recent data shows that the constitutive recycling of claudin-1 is dependent upon the Endosomal Sorting Complex Required for Transport (ESCRT) [10]. The ESCRT machinery, which is made up of ESCRT 0, I, II and III sub-complexes, is required for multiple endocytic trafficking events [15]. ESCRTs have a well established role in the trafficking of transmembrane proteins to the lysosome, but are also required for a number of other processes including, autophagy [16] and endosome to TGN trafficking [17], although this block does not appear to be complete [18]. The ESCRT-III component Vps24/CHMP3, has been shown to bind the phosphoinositide PtdIns(3,5)P2 [19] which is produced from the early endosomally localised lipid PtdIns(3)P by the lipid kinase PIKfyve [20], [21]. The enzyme PIKfyve and its lipid product, like the ESCRT machinery, have been implicated in the endosome to lysosome pathway [22], autophagy [23] and endosome to TGN trafficking [24]. PIKfyve has also been linked to tumour invasion [25], insulin stimulated translocation of the glucose transporter GLUT4 [26], replication of salmonella [27] and regulation of glutamate transporters [28].

Here we address whether addition of a small molecule inhibitor (YM201636) of PIKfyve [29] to epithelial MDCK cells perturbs tight junctions. Our data shows that YM201636 inhibits the constant recycling of claudin-1 and causes it to accumulate intracellularly. In contrast the localisation of ZO-1, occludin and E-cadherin appeared unchanged. We then examined if other claudins behaved like claudin-1. Claudin-2 was found to be constantly endocytosed and recycled in a similar way to claudin-1. Treatment with YM201636 inhibited claudin-2 recycling and caused it to accumulate intracellularly. In contrast clauin-4 showed a much lower rate of endocytosis and YM201636 treatment did not appreciably change the localisation of this protein, arguing that different claudin proteins show different flux through the endocytic system. Finally, we show that, consistent with the defects in claudin trafficking, addition of YM201636 delayed formation of an epithelial permeability barrier. In summary, addition of YM201636 blocked the continuous recycling of claudin-1 and claudin-2 and delayed barrier formation in epithelial cells. To the best of our knowledge this is the first small molecule inhibitor that has been shown to block the recycling of these tight junction proteins.

## Results

### Claudin-1 accumulates intracellularly after treating MDCK cells with YM201636

**Figure 1:**
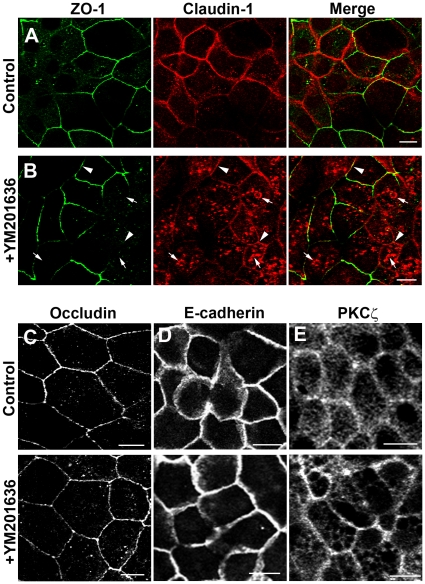
Addition of the PIKfyve inhibitor YM201636 results in intracellular accumulation of claudin-1. YM201636 treated MDCK cells showed no difference in localisation of ZO-1, occludin, E-cadherin or PKCι/ζ when compared with vehicle treated control cells (A–B left panels and C–E). In contrast, the localisation of claudin-1 was dramatically affected by YM201636 treatment, resulting in extensive internal accumulation of the protein (B, middle panel) compared with vehicle control (A, middle panel). Arrows highlight internal claudin-1. Arrowheads highlight claudin-1 that remained at the junctions. Scale bars 10 µm.

MDCK cells were treated with the small molecule inhibitor of PIKfyve (YM201636) [29] and stained for a range of junctional proteins (Figure 1). A dramatic accumulation of claudin-1 on internal structures of cells treated with YM201636 was observed (Figure 1B, arrows). The accumulation of internal caludin-1 coincided with a reduction in plasma membrane staining, however some claudin-1 appeared to remain at the plasma membrane (Figure 1B, arrowheads) so not all claudin-1 relocalised intracellularly. In contrast, localization of the junctional proteins ZO-1, occludin and E-cadherin appeared unaffected by the addition of YM201636 (Figure 1 B, C, D). The localization of the polarity protein aPKCζ/ι also appeared normal after YM201636 treatment (Figure 1E). The accumulation of internal claudin-1 was rapid and increased intracellular claudin-1 could be seen after a 30 minute treatment with YM201636 and extensive accumulation was seen after two hours in a time course experiment (Figure S1).

### YM201636 treatment blocked the constant recycling of claudin-1

We then investigated what trafficking event is being inhibited by YM201636. In order to test the possibility that the accumulation was due to a failure in trafficking of newly synthesised claudin-1 to the plasma membrane, cells were treated with YM201636 in the presence of cyclohexamide to inhibit protein synthesis. Claudin-1 accumulation was still observed (Figure S1B), suggesting that it was not newly synthesised protein but endocytosed claudin-1 that was accumulating.

**Figure 2:**
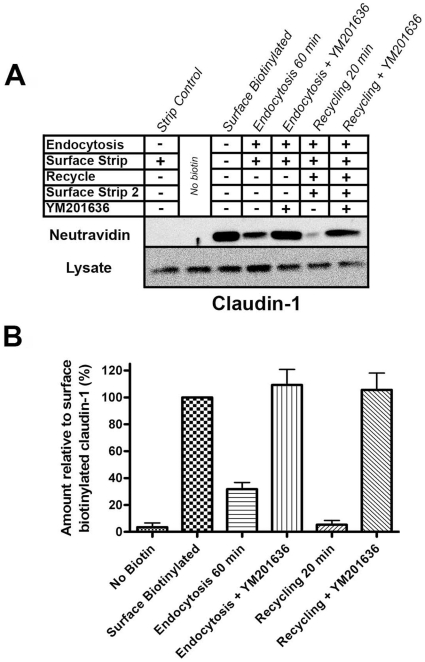
YM201636 blocks the recycling of claudin-1. A surface biotinylation assay was performed on control or YM201636 treated MDCK cells. (A) The ‘Surface Biotinylated’ lane represents the initial biotinylated claudin-1 at the cell surface. Labelled claudin-1 that is internal after 60 min and is resistant to surface stripping is represented for control (‘Endocytosis 60 min’) and YM201636 treated (‘Endocytosis + YM201636) cells. Claudin-1 remaining internal (and thus not recycled back to the surface) following recycling for 20 min is represented as ‘Recycling 20 min’ for control and ‘Recycling + YM201636’ for treated cells. The recycled cargo is then determined as the reduction in signal in the recycling lanes compared to the endocytosis lanes. Addition of YM201636 blocked claudin-1 recycling and caused accumulation of endocytosed protein. Our previous study shows that degradation of claudin-1 does not occur over this timeframe [10] so a block in degradation does not account for the accumulation of internal claudin-1. (B) Results represented graphically show the mean from 3 independent experiments, error bars are SEM.

Claudin-1 is known to be constantly endocytosed and recycled in MDCK cells [10]. In contrast no degradation occurs over the time course of these experiments, so the build up of intracellular protein could not be caused by a block in degradation. To determine whether inhibition of PIKfyve altered the normal endocytosis and recycling of cell surface claudin-1 the biotinylation assay described previously [10], [11] was used. In control cells 35% of the surface labelled claudin-1 was internalised after 60 min (Figure 2, “Endocytosis 60 min”). Proteins that are recycled back to the plasma membrane become accessible to stripping reagent so recycling is shown by a reduction in signal in the “Recycling 20 min” lane compared to the “Endocytosis 60 min” lane (Figure 2). In control cells the majority of the internalised claudin-1 underwent recycling back to the surface after an additional 20 minute incubation at 37°C. The amount of endocytosis and recycling of claudin-1 is consistent with our previous work [10]. In contrast, in cells treated with YM201636 all of the surface biotinylated claudin-1 was internalised after 60 minutes (Figure 2, “Endocytosis + YM201636”). In addition, when cells were treated with YM201636 none of the internalised claudin-1 was returned to the plasma membrane following the second incubation (Figure 2, “Recycling + YM201636”). This result indicates that YM201636 treatment blocked the constitutive recycling of claudin-1, causing an intracellular accumulation of the endocytosed protein.

### Claudin-2 is constantly recycled in MDCK cells and this recycling is blocked by YM201636

**Figure 3:**
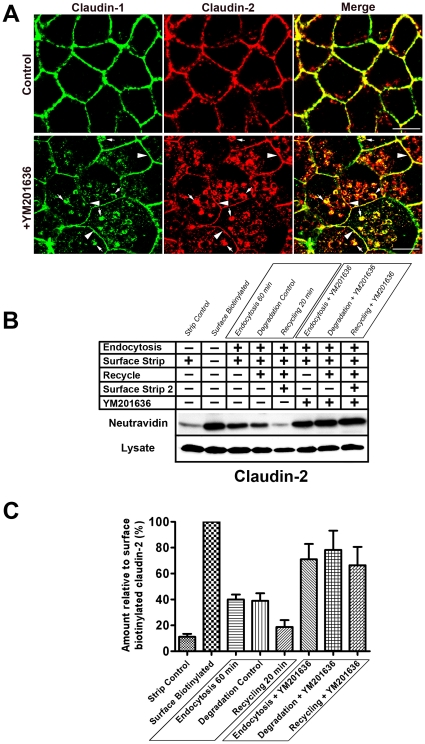
Claudin-2 is constantly recycled in MDCK cells and this recycling is inhibited by YM201636. (A) MDCK cells treated with YM201636, but not DMSO controls, showed accumulation of internal claudin-2 which colocalised with the internal claudin-1 (arrows). Some claudin-2 remained at the junctions (arrowheads). Scale bars 10 µm. (B) A surface biotinylation assay was performed on control or YM201636 treated MDCK cells and a Western blot from a representative experiment is shown. (C) Results represented graphically show the mean from four independent experiments, error bars are SEM. Claudin-2 was found to be endocytosed (endocytosis 60 min) and recycled (shown by the reduction in the ‘Recycling 20 min’ lane compared to the ‘Degradation Control’ lane). Degradation would be shown by a reduction in the ‘Degradation Control’ compared to the ‘Endocytosis 60 min’ but the mean from four experiments showed there was no detectable degradation over this time scale (shown graphically in C). Addition of YM201636 inhibited claudin-2 recycling and caused accumulation of endocytosed proteins.

The claudin family contains more than 20 members [30], so to see if the trafficking of other members was affected by treatment with YM201636 MDCK cells were stained with antibodies for claudin-2. This showed striking accumulation of intracellular claudin-2 following treatment with YM201636 (Figure 3A, arrows) although some remained at the junctions (Figure 3A, arrowheads). The claudin-2 protein which did accumulate intracellularly colocalised with claudin-1.

The immunofluorescence suggests that claudin-2 might be undergoing similar recycling to claudin-1. However, previous work has only examined the trafficking of claudin-1 [10] so the biotinylation assay was used to look at the endocytosis, degradation and recycling of claudin-2 (Figure 3B+C). Endocytosis is shown by a signal in the ‘Endocytosis 60 min’ lane, degradation by a reduction in the ‘Degradation Control’ compared to the ‘Endocytosis 60 min’ lane and recycling by a reduction in ‘Recycling 20 min’ compared to the ‘Degradation Control’. Claudin-2 was found to be endocytosed and recycled without detectable degradation in the time frame of these experiments (Figure 3B+C). This trafficking profile is very similar to claudin-1. Addition of YM201636 blocked this recycling and caused an accumulation of endocytosed claudin-2 (Figure 3B+C).

### Claudin-4 does not show intracellular accumulation following YM201636 treatment and has a low rate of endocytosis

**Figure 4:**
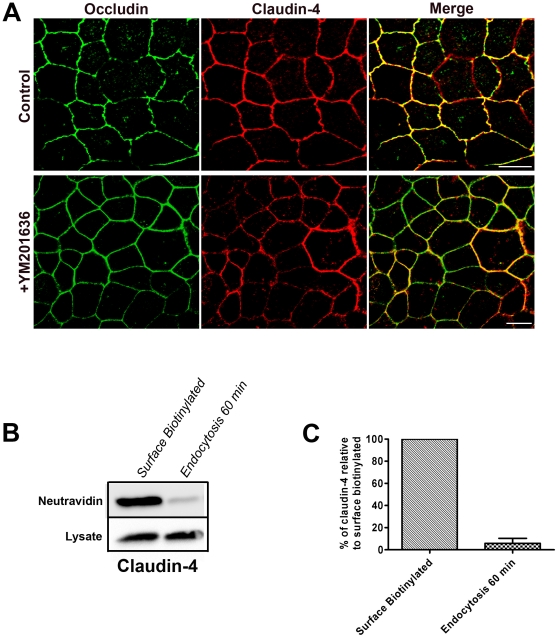
Claudin-4 does not accumulate following YM201636 treatment and undergoes negligible endocytosis. (A) MDCK cells treated with YM201636 showed no detectable accumulation of internal claudin-4. Scale bars 10 µm. (B) A surface biotinylation assay was performed to measure the endocytosis of claudin-4 and a Western blot from a representative experiment is shown. (C) Results represented graphically show the mean from three independent experiments, error bars are SEM. Claudin-4 was successfully biotinylated (Surface Biotinylated) but negligible claudin-4 was internalised (Endocytosis 60 min) suggesting that claudin-4 is endocytosed at a much lower rate than claudin-1 and claudin-2.

Claudin-1 and claudin-2 show a similar response to treatment with YM201636 and have similar profiles of endocytosis and recycling. However, it cannot be assumed that this will apply to all claudins. In fact there was no change in the localisation of claudin-4 following a two hour treatment with YM201636 (Figure 4A).

One explanation for the lack of claudin-4 accumulation is that it is trafficked along a pathway which is not blocked by treatment with YM201636. Alternatively, it might be that the rate of claudin-4 endocytosis is much lower than that seen for claudin-1 and claudin-2. This occurs with occludin, which does not show significant endocytosis after a one hour incubation in MDCK cells [10]. The biotinylation assay was used to measure the endocytosis of claudin-4 and following a one hour incubation negligible internal claudin-4 was present (Figure 4 B+C). Therefore, the rate of claudin-4 endocytosis appears to be much less than for claudin-1 and claudin-2. This demonstrates that there is variation in the rate that claudins are trafficked through the endocytic system and provides a possible explanation for the lack of accumulation seen after addition of YM201636.

### Treatment with YM201636 delays formation of a functional tight junction permeability barrier

**Figure 5:**
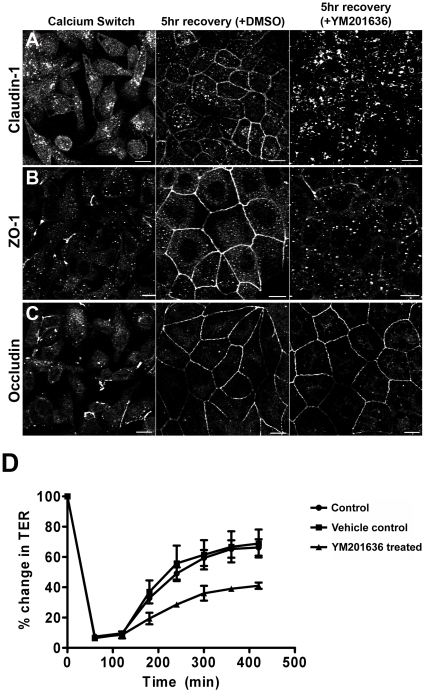
YM201636 impairs tight junction formation following a calcium switch. (A–C) Cells were stained for junctional proteins following calcium removal and showed a loss of staining at the junctions (left panels). Calcium was reintroduced in either control (middle panels) or YM201636 containing media (right panels) and cells were stained for junctional proteins. All proteins showed recovery to the junctions in control conditions. However, following YM201636 treatment claudin-1 and to a lesser extent ZO-1 failed to fully return to the junctions. (D) The transepithelial resistance (TER) of MDCK cells following calcium switch was determined. Treatment with YM201636 caused a delayed recovery of TER. Graphs represent the averages from 3 independent experiments (plus 3 replicates per experiment) and error bars show SEM.

Finally, we investigated whether YM201636 treatment had an effect on the tight junction permeability barrier. Our initial experiments on confluent cells showed no difference between the transepithelial resistance (TER) of YM201636 treated cells and vehicle control treated cells (not shown). We then investigated the establishment of tight junctions using a calcium switch assay [31]. Cells depleted of calcium showed a loss of claudin-1, ZO-1 and occludin staining at the junctions (Figure 5, left panels). In control cells these proteins returned to cell junctions following repletion of calcium (Figure 5, middle panels). When cells were repleted with calcium in the presence of YM201636, occludin returned to cell junctions at a comparable rate to that observed in control cells (Figure 5C, right panel). However, both claudin-1 and ZO-1 failed to return to the junctions in YM201636 treated cells (Figure 5A+B, right panels). A failure of tight junction proteins to efficiently return to the plasma membrane might alter barrier function. To assess this we measured TER following calcium switch and found that cells treated with YM201636 showed a slower rate of recovery of TER in comparison to control cells (Figure 5D). This shows that YM201636 treatment delays the return of tight junction proteins to the plasma membrane and impairs formation of a correct epithelial permeability barrier.

## Discussion

The constant recycling of claudin-1 proteins represents a newly described and poorly understood feature of epithelial cells. In this study we show that claudin-2 is also recycled and that addition of the PIKfyve inhibitor YM201636 interferes with normal claudin-1 and claudin-2 recycling causing accumulation of intracellular claudin proteins. In contrast, in the time frame of these assays claudin-4 underwent negligible endocytosis and the localisation of claudin-4 was not altered by YM201636 treatment. Finally, YM201636 treatment delayed formation of an epithelial permeability barrier, consistent with the alterations in claudin trafficking.

Small molecule inhibitors provide a tractable tool for looking at effects of acutely inhibiting kinase activity and the most likely explanation for our results is that YM201636 is acting by inhibiting PIKfyve. However, the PIKfyve inhibitor may affect multiple targets [32] and to rule out non-specific effects a second structurally distinct inhibitor is required [33]. To our knowledge no such inhibitor is currently available for PIKfyve, so here we conclude that treatment with YM201636 produces the phenotypes described and future work is needed to confirm that YM201636 is functioning via inhibition of PIKfyve.

What is particularly striking about this study was the differences seen between different junction proteins. A very prominent re-localisation of claudin-1 and claudin-2 was seen in the presence of YM201636. In contrast the localisation of other junctional proteins appeared to be indistinguishable from mock-treated cells even after 2 hr of incubation. A similar result was also seen after inhibition of ESCRT function [10]. These results emphasise the different trafficking itineraries that junctional proteins may undertake in epithelial cells. E-cadherin undergoes constitutive internalisation into the endocytic pathway, and has been suggested to traffic through Rab11 positive apical recycling vesicles in MDCK cells [34]. However in this and our previous study on ESCRT proteins [10] we saw no effect on E-cadherin localisation suggesting that E-cadherin and claudins follow distinct recycling pathways. Different tight junction membrane proteins are known to be internalised independently of one another [35], [36]. Furthermore claudin-1 and occludin internalise into different compartments in MDCK cells following calcium depletion [37] and return to the plasma membrane with different kinetics upon calcium repletion [38]. We propose that claudin-1 and claudin-2 follow a distinct trafficking route in epithelial cells that is sensitive to addition of YM201636 and inhibition of ESCRT activity. This pathway is not well defined but may involve Rab13 [38], a GTPase implicated in TGN-endosome or an endosome-TGN retrieval pathway [39]. Interestingly claudin-4 underwent negligible endocytosis in the time frame analysed, suggesting that individual claudins have different rates of flux through the endocytic system.

Previous work used live imaging to look at the dynamics of individual tight junction components over 10–20 minute intervals [9]. This showed that 75% of claudin-1 is stable within the junction while 25% is mobile. Our experiments look at a different aspect of the dynamics of individual tight junction proteins and demonstrate that after an hour approximately 35% of biotinylated claudin-1 is endocytosed ([10]and this work). In addition treatment with YM201636 causes almost 100% of biotinylated claudin-1 to accumulate internally due to the block in recycling. Therefore, the biotin assay suggests that more claudin-1 is mobile than the live imaging methods. A possible explanation for the discrepancy is that the biotinylation is preferentially labelling the claudin-1 which is mobile within the junction, perhaps because it is more accessible than the immobile fraction. This would be consistent with our immunofluorescence data which shows that, despite the large amount of claudin-1 which accumulates internally, some remains at the junctions. If this model is correct then the fraction of junctional claudin which is mobile within the junctions is also rapidly endocytosed and recycled through the endocytic system.

Addition of YM201636 did not alter the TER of confluent monolayers despite causing a striking relocalisation of claudin-1 and claudin-2 to an intracellular pool. This may be due to the fact that some claudin-1 and claudin-2 remained at the junctions. Alternatively it might be because claudin-1 is thought to promote a ‘tight’ seal while claudin-2 is believed to be pore forming [30], [40]. The reduction of one may compensate for the loss of the other. In contrast to the situation in confluent monolayers, following a calcium switch YM201636 treatment reduced the rate of barrier formation. This suggests that formation of an epithelial seal is more sensitive to perturbation with YM201636 than the maintenance of mature junctions. Treatment with YM201636 reduced the amount of claudin-1 which returned to the junctions following a calcium switch, unlike occludin which returned normally. There was also a delay in the return of ZO-1 back to the cell junctions. This was unexpected as there was no effect on ZO-1's localisation in confluent epithelia treated with YM201636. ZO-1 acts as a scaffolding protein that anchors both claudins and occludin to the actin cytoskeleton [41]. We observed some claudin-1 remaining at the junctions after YM201636 treatment and this immobile junctional claudin-1 could anchor ZO-1 at the membrane. In the case of the calcium switch experiments we fail to see any significant claudin-1 returning to the junctions upon calcium repletion, which might explain why ZO-1 is not recruited. This model assumes that the occludin which returns normally after a calcium switch is not sufficient to recruit ZO-1. Future work will need to look at the effects that inhibiting the trafficking of individual junction proteins has on the establishment and maintenance of tight junctions.

In summary, YM201636 treatment causes a block in the endocytic recycling of claudin-1 and claudin-2 and a delay in formation of an epithelial seal. To the best of our knowledge this is the first small molecule inhibitor that has been shown to block the recycling of these tight junction proteins.

## Materials and Methods

### Antibodies and reagents

Rabbit anti-claudin-1 (59–9000), mouse anti-claudin-2 (32–5600), mouse anti-claudin-4 (32–9400), rabbit anti-occludin (71–1500), mouse anti-occludin (33–1500), mouse ZO-1 (33–9100) and mouse E-cadherin (33–4000) were all purchased from Zymed (San Fransico, CA, U.S.A.). Rabbit anti-PKCζ (C-20; sc-216) was obtained from Santa Cruz Biotechnology (Santa Cruz, CA, U.S.A.). Species-specific fluorophore (Alexa Fluor® 546 and 488)-conjugated anti-IgG secondary antibodies were all purchased from Invitrogen. Goat anti-mouse-HRP conjugated secondary antibody was obtained from Sigma and goat anti-rabbit-HRP was purchased from Pierce (Rockford, IL, U.S.A.). All other reagents were purchased from Sigma, unless stated otherwise.

### Cell culture and YM201636 treatment

MDCK type II cells [42] (ECACC, cat no: 00062107) were maintained at 37°C and 5% CO_2_ in DMEM (Dulbecco's modified Eagle's medium) supplemented with 10% (v/v) FBS (fetal bovine serum), 2 mM L-glutamine, 100 units/ml penicillin and 100 µg/ml streptomycin (all from Lonza). Cells were plated onto 13 mm coverslips in 24-well plates (Nunc) and grown 3 d post-confluence prior to treatments. In the case of treatments with YM201636 (and controls), cells were serum starved (complete growth medium lacking FBS) overnight. YM201636 dissolved in DMSO (a kind gift from Professor Peter Shepherd, University of Auckland, New Zealand) was diluted with DMEM and added to cells at a final concentration of 800 nM. Cells were treated with YM201636 or a DMSO control for 2 h unless stated. For TER measurements cells were plated at confluency on Transwell (Corning, Corning, NY, U.S.A., cat no. 3470) permeable polyester filters (0.4 µm pore size) with surface area of 0.33 cm^2^. Media was changed ever 2–3 days and cells were grown for 7 days prior to TER measurements.

### Immunofluorescence

At 24 h post-transfection, cells were fixed with Methanol cooled to -20°C for 10 min. In the case of double staining with anti-claudin-1 and anti-claudin-2, cells were fixed with 4% PFA for 20 min, and then permeabilised with 0.1% TritonX-100 for 15 min. These and other subsequent steps were all performed at room temperature. Cells were then blocked with 10% (v/v) FBS for 30 min. Primary and secondary antibodies were diluted in 2% FBS-PBS (2% FBS in PBS) and cells were incubated with primary antibodies for ∼2 h and ∼1 h for secondary antibodies. Cells were washed five times for 5 min with 2% FBS-PBS following all antibody incubations. Stained cells were then mounted in Mowiol (Calbiochem, San Diego, CA, U.S.A.) and examined on a Zeiss LSM510 laser-scanning confocal microscope and appropriate images taken.

### Endocytosis and recycling biotin assays

The biotinylation assay to study endocytosis and recycling of tight junction proteins was described previously [10], [11]. Briefly, MDCK II cells plated on to 35 mm dishes grown for at least 3 d past confluence and then serum starved over night. Cells were transferred to ice and washed with phosphate buffered saline supplemented with calcium and magnesium (PBS-CM). Cells were then incubated with the cleavable non-membrane permeable sulfo-NHS-SS-biotin (Pierce, cat no: 21331; in PBS-CM) at a concentration of 0.5 mg/ml and incubated with cells for 30 min on ice. Free biotin was then quenched using 50 mM NH_4_Cl (in PBS-CM) for 15 min (4°C). For the endocytosis assay, pre-warmed serum-free medium with either 800 nM YM201636 or DMSO (as a control) was added and cells returned to 37°C for indicated times. Cells were then transferred to ice to stop endocytosis, and surface (non-endocytosed) biotin was stripped by reduction with 100 mM 2-mercaptoethanesulfonate (in tris-buffered saline supplemented with calcium and magnesium; TBS-CM) for 30 min (4°C). Internalised biotinylated cargo was protected from biotin stripping with MESNA by an intact membrane. Free –SH groups were then quenched by incubating cells with 5 mg/ml iodoacetamide (in PBS-CM) for 15 min. For the recycling assay this process was repeated with 20 min incubations at 37°C in serum-free medium supplemented with either YM201636 or DMSO. To control for any loss of biotinylated cargo by degradation, cells were subjected to the full recycling assay in parallel with the recycling condition however the final stripping step was omitted. Thus any loss of biotinylated cargo from this condition would indicate degradation in the 20 min post-endocytosis. To control for the efficiency of surface stripping by MESNA, surface labelled cells were incubated with MESNA as described immediately after surface labelling. Cells were lysed (1.25% (v/v) Triton X-100, 0.25% (w/v) SDS, 50 mM Tris-HCl (pH 8.0), 150 mM NaCl, 5 mM EDTA, 5 mg/ml iodoacetamide, 10 ug/ml APMSF) on ice, pulse sonicated, and spun to remove large/nuclear debris. An equal volume of the post-nuclear supernatant was taken from each sample for use as a loading control. Biotinylated proteins were collected by incubation with Neutravidin beads (Pierce, cat no: 29200,), rotating overnight at 4°C. Beads were then washed by spinning at 1000 g, 5 times with wash buffer (0.5% (v/v) Triton X-100, 0.1% (w/v) SDS, 50 mM Tris-HCl (pH 8.0), 150 mM NaCl, 5 mM EDTA) and 3 times with PBS-CM. Reducing sample buffer was then added to each sample to cleave biotinylated proteins from the beads, and following boiling samples were loaded onto a 15% tris-glycine SDS-PAGE gel. Separated proteins were transferred to nitrocellulose and immunoblotted for the presence of claudin-1 and occludin. Signals were detected by ECL® (enhanced chemiluminescence) or Chemiglow West Chemiluminescence Substrate (Alpha Innotech, San Leandro, CA, USA) and quantified using an Optichem detector with associated software (Ultra Violet Products). For quantification of the biotinylated proteins, the amount of claudin-1 and occludin was normalized to their respective total protein bands determined from non-isolated lysate samples run. Where results were plotted graphically, values were expressed as a percentage of the total claudin-1 biotinylated at the surface only.

### Calcium switch and trans-epithelial resistance (TER)

Media was removed from cells on cover slips or filters (basal and apical chambers), washed once with Dulbecco's phosphate buffered saline (low calcium) and replaced with no calcium essential minimal Eagle's medium. Following 60 min incubation with essential minimal Eagle's medium (37°C) the media was removed and replaced with normal complete growth medium (times indicated) for calcium repletion. For TER studies YM201636 or DMSO was added to both apical and basal chambers, where appropriate. Measurements of TER were carried out using the EVOM TER machine with an Endohm™ chamber (World Precision Instruments, Sarasota, FL, U.S.A.). Measurements of resistance were taken at 60 min intervals and the percentage change in TER (compared to starting TER measurement) was plotted graphically for analysis.

## Supporting Information

Figure S1 **Intracellular claudin-1 accumulates rapidly and is not blocked by addition of cyclohexamide.** (A) YM201636 treatment results in rapid claudin-1 accumulation in MDCK cells. MDCK cells were treated with YM201636 for indicated times (0 min is no treatment), fixed and stained for claudin-1. Increased internal staining of claudin-1 over control (0 min) is observed after 30 min and continues to accumulate over a period of 6 hours. (B) Claudin-1 accumulation is seen in the presence of cyclohexamide. MDCK cells were treated with cyclohexamide (CHX) to inhibit synthesis of new protein and then treated with either DMSO as a vehicle control (left panel) or YM201636 (right panel) for 2 h. Cells were fixed and stained for claudin-1. Scale bars represent 10 µm.
(TIF)
