# Supplementary figures and images for: The PIKfyve Inhibitor YM201636 Blocks the Continuous Recycling of the Tight Junction Proteins Claudin-1 and Claudin-2 in MDCK cells
Source: PLoS One. 2012 Mar 1;7(3):e28659. doi: 10.1371/journal.pone.0028659 (PMC3291620; doi:10.1371/journal.pone.0028659)

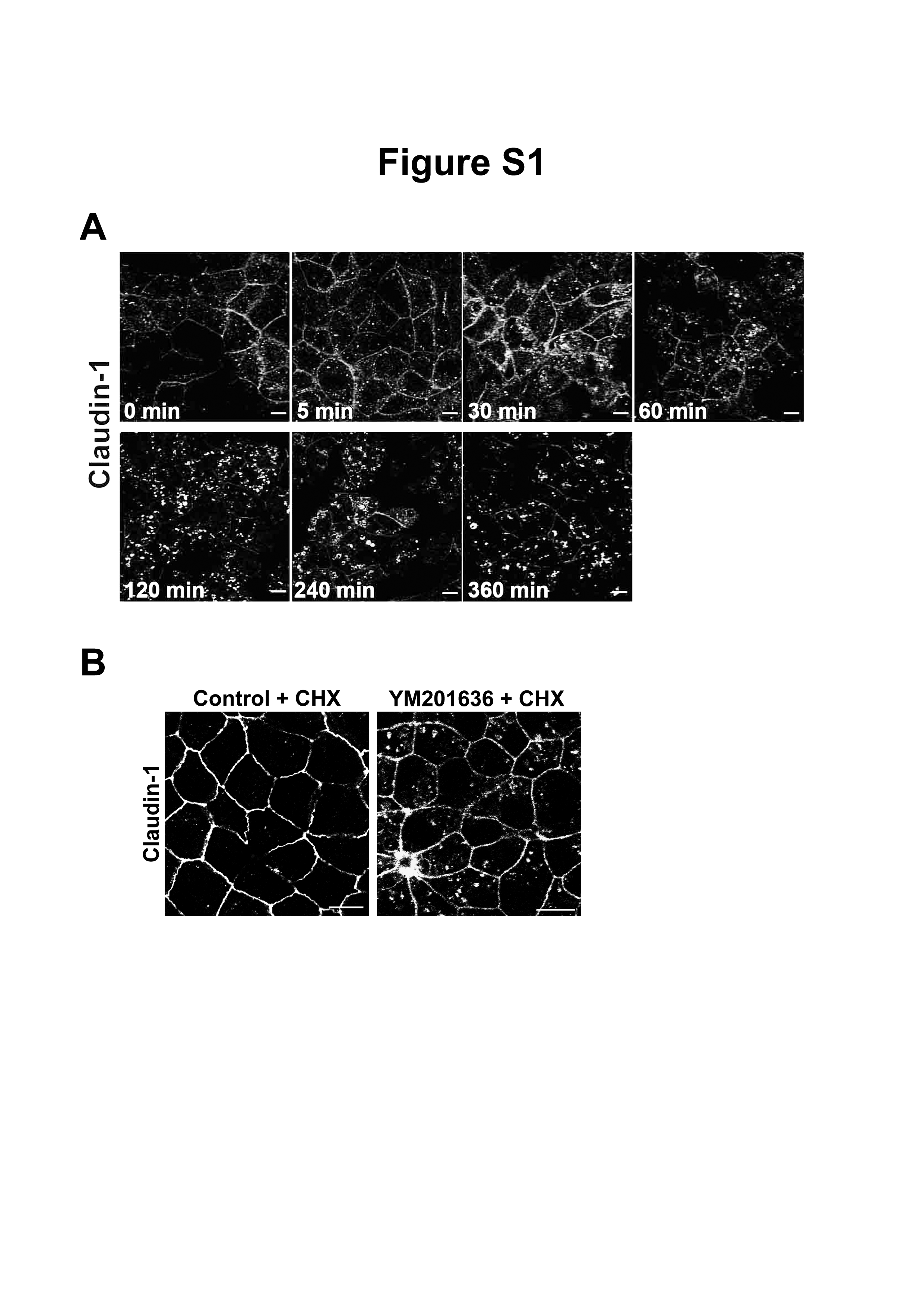

Supplement: Figure S1 — Intracellular claudin-1 accumulates rapidly and is not blocked by addition of cyclohexamide. (A) YM201636 treatment results in rapid claudin-1 accumulation in MDCK cells. MDCK cells were treated with YM201636 for indicated times (0 min is no treatment), fixed and stained for claudin-1. Increased internal staining of claudin-1 over control (0 min) is observed after 30 min and continues to accumulate over a period of 6 hours. (B) Claudin-1 accumulation is seen in the presence of cyclohexamide. MDCK cells were treated with cyclohexamide (CHX) to inhibit synthesis of new protein and then treated with either DMSO as a vehicle control (left panel) or YM201636 (right panel) for 2 h. Cells were fixed and stained for claudin-1. Scale bars represent 10 µm. (TIF) [file pone.0028659.s001.tif]
